# Passive Underwater Target Tracking: Conditionally Minimax Nonlinear Filtering with Bearing-Doppler Observations

**DOI:** 10.3390/s20082257

**Published:** 2020-04-16

**Authors:** Andrey Borisov, Alexey Bosov, Boris Miller, Gregory Miller

**Affiliations:** 1 Institute of Informatics Problems of Federal Research Center “Computer Science and Control” RAS, 44/2 Vavilova Str., Moscow 119333, Russia; aborisov@frccsc.ru (A.B.); abosov@frccsc.ru (A.B.); 2 Institute for Information Transmission Problems RAS, 19/1 Bolshoy Karetny Per., Moscow 127051, Russia; bmiller@iitp.ru; 3 Kazan Federal University, Institute of Physics, 18 Kremlyovskaya Str., Kazan 420008, Russia

**Keywords:** underwater target tracking, bearing-only measurements, bearing-Doppler measurements, port-starboard ambiguity, nonlinear filtering, conditionally minimax nonlinear filter, machine learning

## Abstract

The paper presents an application of the Conditionally-Minimax Nonlinear Filtering (CMNF) algorithm to the online estimation of underwater vehicle movement given a combination of sonar and Doppler discrete-time noisy sensor observations. The proposed filter postulates recurrent “prediction–correction” form with some predefined basic prediction and correction terms, and then they are optimally fused. The CMNF estimates have the following advantageous features. First, the obtained estimates are unbiased. Second, the theoretical covariance matrix of CMNF errors meets the real values. Third, the CMNF algorithm gives a possibility to choose the preliminary observation transform, basic prediction, and correction functions in any specific case of the observation system to improve the estimate accuracy significantly. All the features of conditionally-minimax estimates are demonstrated by the regression example of random position estimate given the noisy bearing observations. The contribution of the paper is the numerical study of the CMNF algorithm applied to the underwater target tracking given bearing-only and bearing-Doppler observations.

## 1. Introduction

The class of underwater target tracking problems is rapidly widening. This process has been provoked not only by the dramatic growth of the underwater vehicle industry, resulting in a big number of multi-functional vehicles available, but also by the active development of the registration devices and improvement of their usage policies along with advanced methods of statistical data processing. The spectrum of applications is also broad and lies both in the civil [1,2,3,4,5] and military areas [6,7,8,9,10].

Without aiming to write an exhaustive review in the area of the underwater target tracking, we should mention the following works [11,12,13]. The analysis of these comprehensive contributions together with other related literature allows for determining the factors and restrictions which affect the performance in any specific applied tracking problem:

– available sensors configuration and their characteristics,
– the mathematical model of the couple “target dynamics–measurements”,
– requirements to the target state estimates,
– theoretical framework and numerical algorithms, involved into the solution to the tracking problem.

The object of this research is the long-range passive tracking problem of a maneuvering target by stationary multi-static sonar site [11]. This task looks rather challenging. First, in this case, the available observations are the bearings towards the target with or without the Doppler shift of the received signal caused by the radial target movement relative to the sonar. The target range is unobservable in the passive mode, which complicates the tracking significantly. Moreover, the bearing measurements of sonars are considerably less accurate in comparison with radars: angular errors of sonars are typically $1^{{}^{\circ}}$–$3^{{}^{\circ}}$ [14], while those of radars vary in the range of $0.05^{{}^{\circ}}$–$0.3^{{}^{\circ}}$ [15]. Second, the negative feature of the sonar observations is the port-starboard ambiguity. The choice of the correct target position is nontrivial and can be done by various methods [6,16,17]. Third, at the moment of the distant target detection, the statistical uncertainty of the position is huge; this forces applying advanced mathematical methods to make the state estimate quality reasonable. Fourth, usually, the moving target is maneuvering [18], and the parameters of the maneuvers are also subject to estimation.

From the mathematical point of view, the target tracking is a filtering problem. The unobservable state is described by a stochastic differential system (SDS), and this state has to be estimated given the indirect discrete-time nonlinear noisy observations. The optimality criterion is usually the Mean Square (MS) of the state estimate error. For practical realization of the filtering process, the common practice is to apply either nonlinear modifications of the well-known Kalman filtering (KF) algorithm: linearized/extended Kalman filter (LKF/EKF) [19,20,21,22], method of pseudo measurements [23,24,25], unscented Kalman filter (UKF) [26,27], quadrature or cubature Kalman filter [28,29], and numerous versions of the particle filter [30,31]. All these methods have known issues: the nonlinear versions of the KF may diverge in specific settings [32,33,34] or provide the biased estimates [23,24], quadrature, and cubature filters are resource-demanding for high-dimensional observation systems [28], and so are the particle filters.

The aim of this paper is the presentation of the Conditionally-Minimax Nonlinear Filtering (CMNF) algorithm for underwater target tracking in the conditions stated above. This algorithm was initially proposed for the state filtering of the discrete-time stochastic finite-dimensional [35] and distributed [36] observation systems. Recently, it was successfully applied to the tracking of a maneuvering aerial target given the radar observations [37] and the navigation of the Automated Underwater Vehicle (AUV) [38].

The idea of the CMNF algorithm is quite transparent. We fix the structure of the filter in the two-step “prediction–correction” form. The transform of the estimate on the previous step (so-called basic prediction function) and the observations on the present step (observation transform and basic correction function) are predefined. The optimization procedure deals only with optimal fusion of these functions in the final filtering estimate in order to provide the best performance in the MS-sense. The minimax property of this fusion is related closely with the one of the pair “Gaussian distribution— linear estimator”. If the mutual distribution of the triplet “state–prediction term–correction term” is uncertain with known mean and covariance matrix bounded from above, then the least favorable distribution is Gaussian, and then the best (equalizing) estimate is the linear one [35,36].

The CMNF estimate has remarkable properties. First, it is unbiased, i.e., the CMNF estimate error is a centered random vector. Second, the CMNF algorithm allows for calculating the upper bound of the error covariance matrix, and this conservative choice guarantees that the algorithm is non-divergent in contrast with the EKF and UKF. Third, the CMNF algorithm gives a possibility to select the functions of basic prediction, observation transform, and correction in accordance with additional a priori information, such as the explicit form of the state dynamics, some guess concerning the form of the optimal nonlinear filter for the observation system at hand, etc. It turns out that a successful choice provides a significant gain in estimation accuracy.

Another aim of the research is to analyze the contribution of Doppler observations into the CMNF performance in comparison with the bearing-only observation version.

The paper is organized as follows. Section 2 contains a description of the target tracking problem. We present the stochastic dynamic system, which defines the target motion, and the model of the sensor measurements.

Section 3 is devoted to the theoretical background of the conditionally-minimax estimation. Traditionally, the key features and performance of the nonlinear KF algorithms like EKF, UKF, etc. are illustrated by a regression example, hence Section 3.1 contains the formulation and solution for the minimax estimation problem of the regression parameters. The mutual distribution of the estimated and observable components is uncertain: their mathematical expectation is known, and the uncertainty set of covariance matrices has a maximal element. An assertion establishing the pair “Gaussian distribution–linear estimator” as a saddle point of the optimality criterion at hand is provided. If the observable part is complemented by some additional transforms of the observations, then it is possible to outperform the accuracy of the best linear estimate. This feature is demonstrated in the regression example. In Section 3.2, we present a conditionally-minimax filtering problem statement for the stochastic continuous-discrete observation systems along with its solution.

Section 4 is the main part of the paper: it contains the comparative material of two numerical experiment groups. Section 4.1 is devoted to the estimation of the three-dimensional random vector given noisy bearing observations. We compare the performance of the best linear and conditionally-minimax estimates. The last involves the least-squares (LS) estimates of the estimated vector as additional pseudo observations. Section 4.2 contains a comparison of the tracking results of a maneuvering target by the noisy bearing-Doppler observations. The maneuver being a planar stochastic coordinate turn with unobservable random target speed and oscillating normal acceleration is described by 17 equations. The parameters of the maneuver plane are also random and unobservable. The concluding remarks are presented in Section 5.

## 2. Model Description and Tracking Problem Formulation

### 2.1. Target Dynamics

**Figure 1:**
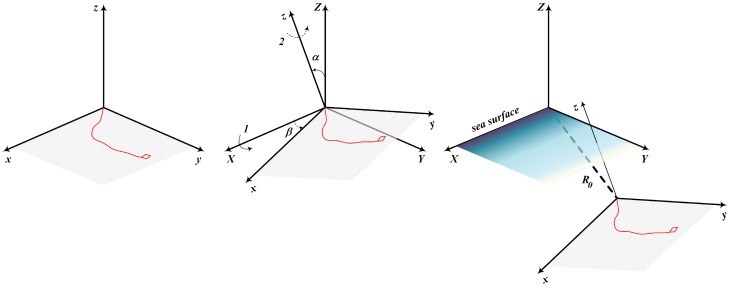
Target and observer coordinate systems.

In what follows, we will operate two coordinate systems. The first one is the coordinate system in which the target motion can be defined as a planar maneuver, and the second one is the coordinate system in which the observations are collected and the estimate is calculated. We call the former “target coordinate system” 0xyz and the latter—“observer coordinate system” 0XYZ. Both systems are stationary, and the translation from one system to another is given by two Euler angles α and β and the shift vector $R^{0}$ (see Figure 1).

The planar target motion in the target coordinate system 0xyz is defined by the following SDS:

(1) $$\left\{\begin{array}{c} dx_{t}=v_{t}\cos\varphi_{t}dt,\\ dy_{t}=v_{t}\sin\varphi_{t}dt,\\ dz_{t}=0,\\ dv_{t}=0,\\ d\varphi_{t}=\frac{a_{t}^{n}}{v_{t}}dt,\\ da_{t}^{n}=\left(-\lambda a_{t}^{n}+\nu \right)dt+\mu dW_{t}, \end{array}\right.$$

where $x_{t}$, $y_{t}$, $z_{t}$ are the coordinates, $v_{t}$ is the absolute value of the target speed, which is constant, $\varphi_{t}$ is the heading angle, $a_{t}^{n}$ is the normal acceleration, and $W_{t}$ is a standard Wiener process. The acceleration equation parameters λ and ν, as well as the disturbance intensity μ, are assumed to be known. In SDS (1), the equations for $x_{t}$, $y_{t}$, $v_{t}$, and $\varphi_{t}$ follow from the standard planar curvilinear-motion kinematic model for constant heading speed motion with the normal acceleration $a_{t}^{n}$, whose dynamics are given by the last equation. In [18], this model is described in detail (see Equations (55), (57)–(60)); the only difference is that here we use the stochastic differential notation (and hence the Wiener process instead of the white Gaussian noise).

The target starts from the origin, so the initial condition for the coordinates is ${\left(x_{0},y_{0},z_{0}\right)}^{T}={\left(0,0,0\right)}^{T}$, but other initial parameters $v_{0}$, $\phi_{0}$, $a_{0}^{n}$ are assumed to be random with known distributions.

In the observer coordinate system, the target has the coordinates $X_{t}={\left(X_{t},Y_{t},Z_{t}\right)}^{T}$ and the vector speed $V_{t}={\left(V_{t}^{X},V_{t}^{Y},V_{t}^{Z}\right)}^{T}$, which are calculated from the target motion parameters $x_{t}={\left(x_{t},y_{t},z_{t}\right)}^{T}$, $v_{t}$, and $\varphi_{t}$ from SDS (1) by the following rules:

(2) $$\left(\begin{array}{c} X_{t}\\ V_{t} \end{array}\right)=\left(\begin{array}{cc} A & 0\\ 0 & A \end{array}\right)\left(\begin{array}{c} x_{t}\\ v_{t} \end{array}\right)+\left(\begin{array}{c} R^{0}\\ 0 \end{array}\right),$$

where $v_{t}={\left(v_{t}\cos\varphi_{t},v_{t}\sin\varphi_{t},0\right)}^{T}$ and A is the rotation matrix

$$A=\left(\begin{array}{ccc} \cos\alpha & -\sin\alpha \cos\beta & \sin\alpha \sin\beta \\ \sin\alpha & \cos\alpha \cos\beta & -\cos\alpha \sin\beta \\ 0 & \sin\beta & \cos\beta \end{array}\right),$$

The parameters of the target coordinate system rotation α, β, and shift $R^{0}$ are assumed to be unknown random values with known distributions. These parameters are subject to estimation, and, to that end, we introduce the following auxiliary dynamic equations:

(3) $$\left\{\begin{array}{c} d\alpha_{t}=0,\\ d\beta_{t}=0,\\ dR_{t}^{0}={\left(dR_{0}^{X}\left(t\right),dR_{0}^{Y}\left(t\right),dR_{0}^{Z}\left(t\right)\right)}^{T}=0 \end{array}\right.$$

with initial conditions $\alpha_{0}=\alpha$, $\beta_{0}=\beta$, $R_{0}^{0}=R^{0}$.

Summing up the planar target dynamic Equation (1), the coordinate transform Equation (2), and the equations for the auxiliary variables (3), we have a 17-dimensional stochastic differential system.

### 2.2. Observations

The target is observed by a set of *N* registration devices (acoustic sensors) which measure the bearing and the Doppler frequency shift. The coordinates $\left\{X^{i}\right\}=\left\{{\left(X^{i},Y^{i},Z^{i}\right)}^{T}\right\}$, i=1,…,N of these devices are assumed to be known, as well as the precision of the measurements they provide. The observations are available at known time instants $t_{k}$, k=0,…,K of the observation interval t∈[0,T] with $t_{0}=0$ and $t_{K}=T$. For the sake of simplicity, we presume equal intervals between the observations: $\Delta =t_{k+1}-t_{k}$, k=0,…,K−1. The observable bearing is connected with the target motion parameters by the following relations:

(4) $$\left\{\begin{array}{c} \xi_{t_{k}}^{i}=\frac{Z_{t_{k}}-Z^{i}}{R_{t_{k}}^{i}}+v_{k}^{\xi^{i}},\\ \eta_{t_{k}}^{i}=\frac{X_{t_{k}}-X^{i}}{r_{t_{k}}^{i}}+v_{k}^{\eta^{i}}, \end{array}\right.$$

where $R^{i}=∥R^{i}∥$ is the range from the *i*-th observer to the target, and $r^{i}$ is the length of the position vector $R^{i}$ projection on the plane Z=0:

$$\begin{array}{c} R_{t_{k}}^{i}=\sqrt{{\left(X_{t_{k}}-X^{i}\right)}^{2}+{\left(Y_{t_{k}}-Y^{i}\right)}^{2}+{\left(Z_{t_{k}}-Z^{i}\right)}^{2}},\\ r_{t_{k}}^{i}=\sqrt{{\left(X_{t_{k}}-X^{i}\right)}^{2}+{\left(Y_{t_{k}}-Y^{i}\right)}^{2}}. \end{array}$$

**Figure 2:**
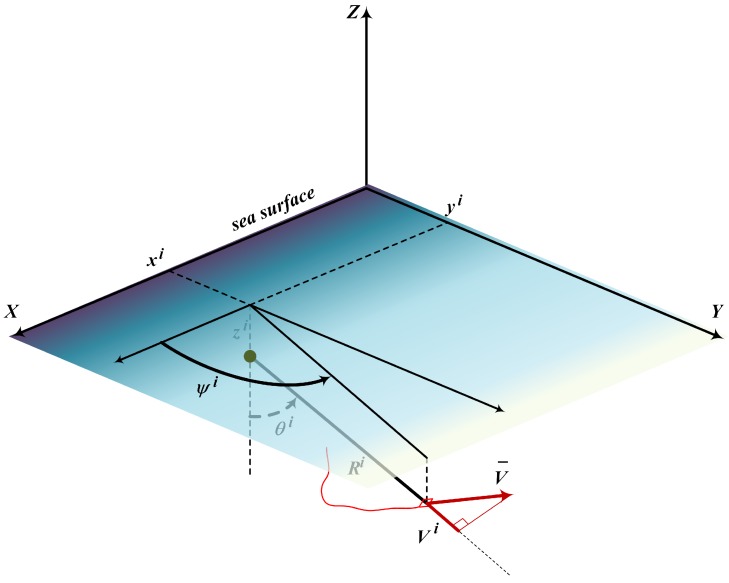
Bearing observations of the target from the *i*-th observer.

The observation system is presented in detail in Figure 2. As one can see, the observations $\xi^{i}=\cos\psi^{i}$ and $\eta^{i}=\cos\theta^{i}$ are the direction cosines of $R^{i}$. Note that this structure of observations presumes the presence of the port-starboard ambiguity.

The Doppler measurements bring information concerning the radial target speed towards the sensor. The Doppler data processing makes the accuracy of the target state estimate significantly better. In the case of active tracking, the target is irradiated by an emitter with a known fixed frequency, and the receiver compares a frequency of the reflected signal with the original. In the case of passive observations, tracking becomes a challenging problem. Only the acoustic noise (so-called target acoustic signature) distorted by the target motion relative to the sensor is available for observation. The signature identification itself is an individual non-trivial problem [39,40,41,42,43,44] outside the scope of our study. To simplify the presentation, we suppose that the acoustic signature is known and determined by some known fixed frequency $\omega_{0}$, whose Doppler distortions we can register.

Thus, the Doppler shift measurements are given by the following expression:

(5) $$\omega_{t_{k}}^{i}=\frac{\omega_{0}}{1-V_{t_{k}}^{i}/C}+v_{k}^{\omega^{i}},$$

where $\omega_{0}$ is the frequency of sound emitted by the target, $\omega^{i}$ is the frequency registered by the observer *i*, *C* is the sound speed in the medium, and $V_{t_{k}}^{i}$ is the relative speed of the target with respect to the observer *i*:

$$V_{t_{k}}^{i}=\frac{V_{t_{k}}^{X}\left(X_{t_{k}}-X^{i}\right)+V_{t_{k}}^{Y}\left(Y_{t_{k}}-Y^{i}\right)+V_{t_{k}}^{Z}\left(Z_{t_{k}}-Z^{i}\right)}{R_{t_{k}}^{i}}.$$

The observation noise ${\left(v_{k}^{\xi^{1}},v_{k}^{\eta^{1}},v_{k}^{\omega^{1}},\ldots ,v_{k}^{\xi^{N}},v_{k}^{\eta^{N}},v_{k}^{\omega^{N}}\right)}^{T}$ is a sequence of independent and identically distributed random vectors with uncorrelated components. The mean values and standard deviations of the noise components are assumed to be known.

### 2.3. Tracking Problem Formulation

Now that the target dynamics and the observation model are defined, the tracking problem can be formulated as filtering, i.e., search for the estimate ${\hat{X}}_{t}={\left({\hat{X}}_{t},{\hat{V}}_{t},{\hat{x}}_{t},{\hat{v}}_{t},{\hat{\phi }}_{t},{\hat{a}}_{t}^{n},{\hat{\alpha }}_{t},{\hat{\beta }}_{t},{\hat{R}}_{t}^{0}\right)}^{T}$ of the target motion parameters

(6) $$X_{t}=X_{0}+\int_{0}^{t}a\left(X_{s}\right)ds+\int_{0}^{t}bd\ell_{s},$$

where $a(X_{s})$ and *b* are defined by SDS (1)–(3) given the bearing-only observations

(7) $$Y_{k}^{b}=\mathrm{col}\left(\left\{{\left(\xi_{t_{k}}^{i},\eta_{t_{k}}^{i}\right)}^{T}\right\}\right),$$

defined by Equation (4), or combined bearing-Doppler observations

(8) $$Y_{k}^{bd}=\mathrm{col}\left(\left\{{\left(\xi_{t_{k}}^{i},\eta_{t_{k}}^{i},\omega_{t_{k}}^{i}\right)}^{T}\right\}\right),$$

defined by Equations (4) and (5), i=1,…,N, k=0,…,K. Here, col(·) operator applied to a set of vectors $\left\{a_{i}\right\}$, i=1,…,N results in vertically stacking of those vectors into one: $\mathrm{col}\left(\left\{a_{i}\right\}\right)={\left(a_{1}^{T},\ldots a_{N}^{T}\right)}^{T}$.

## 3. Basics of Conditionally-Minimax Filtering

This section contains a brief description of the CM framework and the theorems necessary for the filter design [35,36].

### 3.1. Minimax Estimation in Regression

The CMNF algorithm is based on the solution to the following minimax estimation problem. Let us consider a compound random vector $Q≜\mathrm{col}\left(X,Y\right)\in R^{n+m}$, where $X\in R^{n}$ is an unobservable estimated part, and $Y\in R^{m}$ is the observation. The probability distribution P of *Q* is uncertain: the mean $m_{Q}≜E_{P}\left\{Q\right\}=\mathrm{col}\left(m_{X},m_{Y}\right)$ is known, but the covariance $R^{Q}≜{\mathrm{cov}}_{P}\left(Q,Q\right)$ is unknown but bounded:

$$\underline{R}≜\left(\begin{array}{cc} {\underline{R}}^{\mathrm{XX}} & {\underline{R}}^{\mathrm{XY}}\\ {\underline{R}}^{\mathrm{YX}} & {\underline{R}}^{\mathrm{YY}} \end{array}\right)⩽R^{Q}⩽\bar{R}≜\left(\begin{array}{cc} {\bar{R}}^{\mathrm{XX}} & {\bar{R}}^{\mathrm{XY}}\\ {\bar{R}}^{\mathrm{YX}} & {\bar{R}}^{\mathrm{YY}} \end{array}\right).$$

Here, $\underline{R},\;\bar{R}\in R_{+}^{\left(n+m)\times (n+m\right)}$ are known positively semidefinite matrices and matrix inequality A⩽B means that the matrix B−A is positive semidefinite. The set of all feasible distributions P is denoted by P. All functions $\psi :\;R^{m}\to R^{n}$ such that $\underset{P\in P}{\sup}E_{P}\left\{{∥\psi \left(Y\right)-X∥}^{2}\right\}<+\infty$ form the class of admissible estimators Ψ. The performance of an estimator ψ(·) given the distribution P is determined by the traditional MS-criterion $J\left(P,\psi \right)≜E_{P}\left\{{∥\psi \left(Y\right)-X∥}^{2}\right\}$.

The minimax estimation problem of X given Y is to find $\hat{X}≜\hat{\psi }\left(Y\right)$ such that

$$\hat{\psi }\left(\cdot \right)\in \underset{\psi \in \Psi }{\mathrm{Argmin}}\underset{P\in P}{\sup}J\left(P,\psi \right).$$

**Theorem** **1.** *The minimax estimate $\hat{X}$ is linear*

(9) $$\hat{X}=\hat{\psi }\left(X\right)≜m_{X}+{\bar{R}}^{\mathrm{XY}}{\left({\bar{R}}^{\mathrm{YY}}\right)}^{+}\left(Y-m_{Y}\right),$$

*where $A^{+}$ denotes matrix pseudo-inverse. On the set P×Ψ, the criterion J has saddle points, i.e.,*

$$\min_{\psi \in \Psi }\max_{P\in P}J\left(P,\psi \right)=\max_{P\in P}\min_{\psi \in \Psi }J\left(P,\psi \right).$$

*The Gaussian distribution $N(m_{Q},\bar{R})$ with the expectation $m_{Q}$ and the maximal feasible covariance matrix $\bar{R}$ is the least favorable one, and the pair $\left(N\left(m_{Q},\bar{R}\right),\hat{\psi }\right)$ is one of the saddle points. Hence, the double inequality*

$$J\left(P,\hat{\psi }\right)⩽J\left(N\left(m_{Q},\bar{R}\right),\hat{\psi }\right)⩽J\left(N\left(m_{Q},\bar{R}\right),\psi \right)$$

*holds for all (P,ψ)∈P×Ψ.*

*The estimate $\hat{X}$ has guaranteeing accuracy:*

$$E\left\{\left(\hat{X}-X\right){\left(\hat{X}-X\right)}^{⊤}\right\}⩽{\bar{R}}^{\mathrm{XX}}-{\bar{R}}^{\mathrm{XY}}{\left({\bar{R}}^{\mathrm{YY}}\right)}^{+}{\bar{R}}^{\mathrm{YX}},$$

*and the equality holds for any P∈P with ${\mathrm{cov}}_{P}\left(Q,Q\right)=\bar{R}$.*

The proof of Theorem 1 is given in [35,36]. The theorem plays a key role in the development of the CMNF algorithm. First, it guarantees the estimate unbiasedness and gives a possibility to calculate a conservative approximation of the accuracy characteristic, adequate to reality. Second, the minimax estimate can be obtained under a priori uncertainty of probability distribution. To calculate the minimax estimate, it is sufficient to know the mean and some upper bound of the covariance. The closer the obtained upper bound is to the real covariance, the closer the conservative accuracy approximation is to reality. Third, the extension of the admissible estimators by the nonlinear functions given initial observations only does not enhance the estimation quality. Fourth, since the least favorable distribution is Gaussian, the moments of the 1st and 2nd order form a closed set of sufficient statistics in the problem, and, at the same time, it gives a certain justification of usage of the Gaussianity assumption concerning both the prediction and correction terms in the EKF and UKF. In fact, estimate (9) is the best linear one for the least favorable distribution $N(m_{Q},\bar{R})$. Note that the regression estimates calculated either via statistical linearization method or UT-transform, being affine functions of the observations, can be considered as some approximations of the minimax estimate $\hat{X}$: terms $m_{X}$ and $m_{Y}$ along with the gain matrix ${\bar{R}}^{\mathrm{XY}}{\left({\bar{R}}^{\mathrm{YY}}\right)}^{+}$ are replaced in Formula (9) by some estimates of *fixed precision*. The term “fixed precision” means that there is no way of asymptotic approximation of $m_{X}$, $m_{Y}$ and ${\bar{R}}^{\mathrm{XY}}{\left({\bar{R}}^{\mathrm{YY}}\right)}^{+}$ neither by the statistical linearization nor by the UT-transform. By contrast, the CMNF algorithm uses the Monte Carlo method and gives a possibility to calculate coefficients with arbitrary precision.

The key advantage of the CM framework is the possibility to construct the estimate, which is better than the optimal linear estimate (9), amplifying the initial observation Y with some transform. Let us introduce a function $z=z\left(y\right):\;R^{m}\to R^{k}$, such that $\sup_{P\in P}E_{P}\left\{{∥z\left(Y\right)∥}^{2}\right\}<\infty$ and the corresponding compound vector $Q^{\prime }≜\mathrm{col}\left(X,Y,z\left(Y\right)\right)$. Again, the probability distribution $P^{\prime }$ of $Q^{\prime }$ is supposed to be uncertain: the mean $m_{Q^{\prime }}≜E_{P^{\prime }}\left\{Q^{\prime }\right\}=\mathrm{col}\left(m_{X},m_{Y},m_{Z}\right)$ is known, the covariance $R^{Q^{\prime }}≜{\mathrm{cov}}_{P}\left(Q^{\prime },Q^{\prime }\right)$ is unknown but bounded:

$$\underline{R}≜\left(\begin{array}{ccc} {\underline{R}}^{\mathrm{XX}} & {\underline{R}}^{\mathrm{XY}} & {\underline{R}}^{\mathrm{XZ}}\\ {\underline{R}}^{\mathrm{YX}} & {\underline{R}}^{\mathrm{YY}} & {\underline{R}}^{\mathrm{YZ}}\\ {\underline{R}}^{\mathrm{ZX}} & {\underline{R}}^{\mathrm{ZY}} & {\underline{R}}^{\mathrm{ZZ}} \end{array}\right)⩽R^{Q^{\prime }}⩽\bar{R}≜\left(\begin{array}{ccc} {\bar{R}}^{\mathrm{XX}} & {\bar{R}}^{\mathrm{XY}} & {\bar{R}}^{\mathrm{XZ}}\\ {\bar{R}}^{\mathrm{YX}} & {\bar{R}}^{\mathrm{YY}} & {\bar{R}}^{\mathrm{YZ}}\\ {\bar{R}}^{\mathrm{ZX}} & {\bar{R}}^{\mathrm{ZY}} & {\bar{R}}^{\mathrm{ZZ}} \end{array}\right).$$

Here, $\underline{R},\;\bar{R}\in R_{+}^{\left(n+m+k)\times (n+m+k\right)}$ are known positively semidefinite matrices, including the lower and upper bounds $\underline{R}$ and $\bar{R}$ as the corresponding block components. The set of all feasible distributions $P^{\prime }$ is denoted by $P^{\prime }$. All functions $\psi^{\prime }:\;R^{m+k}\to R^{n}$ such that $\sup_{P^{\prime }\in P^{\prime }}E_{P^{\prime }}\left\{{∥\psi \left(Y,z\left(Y\right)\right)-X∥}^{2}\right\}<+\infty$ form the class of admissible estimators $\Psi^{\prime }$. The performance of estimator $\psi^{\prime }\left(\cdot \right)$ given the distribution $P^{\prime }$ is determined as $J\left(P^{\prime },\psi^{\prime }\right)≜E_{P^{\prime }}\left\{{∥\psi \left(Y,z\left(Y\right)\right)-X∥}^{2}\right\}$. In view of Theorem 1, the minimax estimate ${\hat{X}}^{\prime }$ given col(Y,z(Y)) takes the form

$${\hat{X}}^{\prime }=m_{X}+\left(\begin{array}{cc} {\bar{R}}^{\mathrm{XY}} & {\bar{R}}^{\mathrm{XZ}} \end{array}\right){\left(\begin{array}{cc} {\bar{R}}^{\mathrm{YY}} & {\bar{R}}^{\mathrm{YZ}}\\ {\bar{R}}^{\mathrm{ZY}} & {\bar{R}}^{\mathrm{ZZ}} \end{array}\right)}^{+}\left(\left(\begin{array}{c} Y\\ z(Y) \end{array}\right)-\left(\begin{array}{c} m_{Y}\\ m_{Z} \end{array}\right)\right),$$

and its guaranteeing accuracy is defined by the inequality:

$$E\left\{\left({\hat{X}}^{\prime }-X\right){\left({\hat{X}}^{\prime }-X\right)}^{⊤}\right\}⩽{\bar{R}}^{\mathrm{XX}}-\left(\begin{array}{cc} {\bar{R}}^{\mathrm{XY}} & {\bar{R}}^{\mathrm{XZ}} \end{array}\right){\left(\begin{array}{cc} {\bar{R}}^{\mathrm{YY}} & {\bar{R}}^{\mathrm{YZ}}\\ {\bar{R}}^{\mathrm{ZY}} & {\bar{R}}^{\mathrm{ZZ}} \end{array}\right)}^{+}\left(\begin{array}{c} {\bar{R}}^{\mathrm{YX}}\\ {\bar{R}}^{\mathrm{ZX}} \end{array}\right)≜\hat{R}.$$

From the properties of nonnegatively semidefinite matrix pseudo-inverse, it follows that $\hat{R}⩽\hat{R}$ for any admissible z(y), i.e., taking into account any admissible observation transform enhances the estimation performance. The effective choice of an appropriate transform looks like some sort of art, and it should be done reasoning from the properties of a specific observation system at hand.

### 3.2. CMNF in Continuous-Discrete Systems

On the probability triplet with filtration $\left(\Omega ,F,P,\left\{F_{t}\right\}\right)$, we consider the observation system

$$\left\{\begin{array}{c} X_{t}=X_{0}+\int_{0}^{t}a\left(s,X_{s}\right)ds+\int_{0}^{t}b\left(s,X_{s-}\right)d\ell_{s},\;t>0,\\ Y_{k}=c\left(t_{k},X_{t_{k}},v_{k}\right),\;t_{k+1}-t_{k}=\Delta ,\;k\in N. \end{array}\right.$$

Here,

– $X_{t}\in R^{n}$ is an unobservable state process subject to estimation with an initial condition $X_{0}$,
– $\ell_{t}\in R^{n}$ is an $F_{t}$-adapted square-integrable Levy process,
– $Y_{k}\in R^{m}$ is the observation sequence, available at the equidistant instants $t_{k}$ with the step Δ,
– $v_{k}\in R^{m}$ is a $F_{t_{k}}$-adapted sequence of i.i.d. random vectors; $x_{0}$, $\left\{\ell_{t}\right\}$ and $\left\{v_{k}\right\}$ are supposed to be mutually independent.

We assume for the drift a(·,·) and diffusion b(·,·) the standard conditions guaranteeing the existence and uniqueness of a strong solution to the stochastic differential equation for $X_{t}$ along with finiteness of its moments [45]. The observation function c(·,·,·) is also assumed to provide the existence of the moments up to order 2 for the observations $Y_{k}$.

Generally, the problem is to find an estimate ${\tilde{X}}_{k}$ of the state $X_{k}=X_{t_{k}}$, given the available observations up to the moment $t_{k}$. The estimation quality is characterized by a traditional MS-criterion.

The filter structure is partially determined by three sequences of functions $\left\{\alpha_{k}\right\}$, $\left\{\beta_{k}\right\}$ and $\left\{\gamma_{k}\right\}$:

– $\alpha_{k}\left(x\right):R^{n}\to R^{n}$ is basic prediction function,
– $\beta_{k}\left(y\right):R^{m}\to R^{k}$ is observation transform,
– $\gamma_{k}\left(x,z\right):R^{n}\times R^{k}\to R^{k}$ is basic correction function.

The admissible filter has the following recursive form

(10) $${\tilde{X}}_{k}=\phi_{k}\left(\alpha_{k}\left({\tilde{X}}_{k-1}\right),\gamma_{k}({\tilde{X}}_{k-1},\beta_{k}\left(Y_{k})\right)\right),$$

where the sequence $\left\{\phi_{k}\right\}$ of functions $\phi_{k}\left(x,z\right):R^{n}\times R^{k}\to R^{n}$ should be optimized. It is supposed that the functions $\alpha_{k}\left(\cdot \right)$, $\beta_{k}\left(\cdot \right)$ and $\gamma_{k}\left(\cdot \right)$ of the corresponding random arguments in Formula (10) have finite moments up to order 2. The set $\Phi_{k}$ of admissible filters is also formed by all the measurable functions, such that $E\left\{∥{\tilde{X}}_{k}{∥}^{2}\right\}<\infty$.

The conditions above guarantee that the compound random vector

$$Q_{k}≜\mathrm{col}\left(X_{k},\alpha_{k}\left({\tilde{X}}_{k-1}\right),\gamma_{k}\left({\tilde{X}}_{k-1},\beta_{k}\left(Y_{k}\right)\right)\right)$$

has two moments $m_{k}^{Q}≜E\left\{Q_{k}\right\}$ and $R_{k}^{Q}≜\mathrm{cov}\left(Q_{k},Q_{k}\right)$:

$$m_{k}^{Q}=\left(\begin{array}{c} m_{k}^{x}\\ m_{k}^{\alpha }\\ m_{k}^{\gamma } \end{array}\right),\;R_{k}^{Q}=\left(\begin{array}{ccc} R_{k}^{xx} & R_{k}^{x\alpha } & R_{k}^{x\gamma }\\ R_{k}^{\alpha x} & R_{k}^{\alpha \alpha } & R_{k}^{\alpha \gamma }\\ R_{k}^{\gamma x} & R_{k}^{\gamma \alpha } & R_{k}^{\gamma \gamma } \end{array}\right).$$

Let us denote by $P_{k}$ the set of all probability distributions of (2n+k)-dimensional random vectors with known expectation and unknown covariance matrix bounded from above: $R_{k}^{Q}⩽{\bar{R}}_{k}$ with some known positive semidefinite matrix ${\bar{R}}_{k}$.

The conditionally-minimax filtering problem is to find

$${\hat{\phi }}_{k}\in \underset{\phi_{k}\in \Phi_{k}}{\mathrm{Argmin}}\;\underset{P\in P_{k}}{\sup}\;E_{P}\left\{∥\phi_{k}\left(\alpha_{k}\left({\tilde{X}}_{k-1}\right),\gamma_{k}({\tilde{X}}_{k-1},\beta_{k}\left(Y_{k})\right)\right)-X_{k}{∥}^{2}\right\}.$$

**Theorem** **2.** *The CMNF has a two-step “prediction–correction” form. The prediction and the upper bound of its error covariance matrix are defined as follows:*

(11) $$\begin{array}{c} {\overset{˘}{X}}_{k}={\bar{R}}_{k}^{x\alpha }{\left({\bar{R}}_{k}^{\alpha \alpha }\right)}^{+}\alpha_{k}\left({\hat{X}}_{k-1}\right)+\left[m_{k}^{x}-{\bar{R}}_{k}^{x\alpha }{\left({\bar{R}}_{k}^{\alpha \alpha }\right)}^{+}m_{k}^{\alpha }\right],\\ \mathrm{cov}\left({\overset{˘}{X}}_{k}-X_{k},{\overset{˘}{X}}_{k}-X_{k}\right)⩽{\overset{˘}{K}}_{k}^{xx}≜{\bar{R}}_{k}^{xx}-{\bar{R}}_{k}^{x\alpha }{\left({\bar{R}}_{k}^{\alpha \alpha }\right)}^{+}{\bar{R}}_{k}^{\alpha x}. \end{array}$$

*The correction and the upper bound of its error covariance matrix are defined as follows:*

(12) $$\begin{array}{cc} {\hat{X}}_{k}={\overset{˘}{X}}_{k}+{\overset{˘}{K}}_{k}^{x\gamma }{\left[{\overset{˘}{K}}_{k}^{\gamma \gamma }\right]}^{+}{\overset{˘}{\gamma }}_{k},\; & \mathrm{cov}\left({\hat{X}}_{k}-X_{k},{\hat{X}}_{k}-X_{k}\right)⩽{\hat{K}}_{k}^{xx}≜{\overset{˘}{K}}_{k}^{xx}-{\overset{˘}{K}}_{k}^{x\gamma }{\left({\overset{˘}{K}}_{k}^{\gamma \gamma }\right)}^{+}{\overset{˘}{K}}_{k}^{\gamma x}, \end{array}$$

*where*

$${\overset{˘}{\gamma }}_{k}=\gamma_{k}\left({\hat{X}}_{k-1},\beta_{k}\left(Y_{k}\right)\right)-m_{k}^{\gamma }-{\bar{R}}_{k}^{\gamma \alpha }{\left({\bar{R}}_{k}^{\alpha \alpha }\right)}^{+}\left(\alpha_{k}\left({\hat{X}}_{k-1}\right)-m_{k}^{\alpha }\right)$$

*is the modified correction, and*

$$\begin{array}{cc} {\overset{˘}{K}}_{k}^{x\gamma }≜{\bar{R}}_{k}^{x\gamma }-{\bar{R}}_{k}^{x\alpha }{\left({\bar{R}}_{k}^{\alpha \alpha }\right)}^{+}{\bar{R}}_{k}^{\alpha \gamma },\; & {\overset{˘}{K}}_{k}^{\gamma \gamma }≜{\bar{R}}_{k}^{\gamma \gamma }-{\bar{R}}_{k}^{\gamma \alpha }{\left({\bar{R}}_{k}^{\alpha \alpha }\right)}^{+}{\bar{R}}_{k}^{\alpha \gamma }. \end{array}$$

Proof of Theorem 2 follows immediately from assertions of Theorem 1 and the recursive variant of Theorem 13.1 from [46]. The proposed CMNF has the following positive properties similar to the minimax regression estimates: unbiasedness and conservative approximation of the covariance matrix. Moreover, the CMNF algorithm gives additional possibilities. First, the transformation of observations gives a chance to raise the estimation accuracy. For example, the inclusion of the powers of the original observations into the calculation process allows for computing polynomial estimates of better accuracy than provided by the best linear estimates [47]. Second, the proper choice of the basic prediction and correction functions for each specified observation system also allows for raising the estimation quality to the optimal. For instance, in the case of the linear Gaussian observation system, identical observation transform, linear prediction, and correction, the proposed CMNF turns into the classical KF [48].

There is a considerable issue of CMNF implementation. To calculate CMNF, one needs to know the expectation and covariance matrix (or at least its conservative approximation) of the compound vectors $\left\{Q_{k}\right\}$. The analytical calculation of these characteristics is possible only for a narrow class of observation systems, including linear Gaussian and the ones with Markov jump processes as the states [47]. In general, the calculation of these parameters is possible through numerically expensive Monte Carlo sampling. The approximations of the mean can be obtained by regular sampling; meanwhile, the covariance matrix should be approximated from above, using the asymptotic confidence regions. The desired sample size can be controlled based on the results of [49].

## 4. Comparative Numerical Study

### 4.1. Estimation of Static Cartesian Coordinates Given Bearing-Only Multisensor Observations

In this section, we present the simulation results for the static case, which can be considered as the initial step of the filtering problem for the dynamic system presented in Section 2. All the simulation in Section 4.1 and Section 4.2 was done using Python, the source code is available in public repository [50].

The position of the target is a random vector $X_{0}$ with independent components. The distribution of each component is Gaussian with the following parameters (mean and standard deviation in meters):

(13) $$X_{0}∼N\left(0,10^{3}\right),\;Y_{0}∼N\left(y_{0},10^{3}\right),\;Z_{0}∼N\left(-10^{3},10^{2}\right),$$

where the parameter $y_{0}$ takes values $\left\{5,10,20\right\}\times 10^{3}$. This diversity is introduced in order to demonstrate how the estimates depend on the degree of the nonlinearity of the system at hand.

Four pairs of registration devices (acoustic sonars) are located at the points $\left\{X^{i}\right\}$, i=1,…,8:

(14) $$\begin{array}{cccc} {\left(-10000,0,-25\right)}^{T}, & {\left(-5000,1000,-25\right)}^{T}, & {\left(5000,1000,-25\right)}^{T}, & {\left(10000,0,-25\right)}^{T},\\ {\left(-10000,0,-50\right)}^{T}, & {\left(-5000,1000,-50\right)}^{T}, & {\left(5000,1000,-50\right)}^{T}, & {\left(10000,0,-50\right)}^{T}. \end{array}$$

The observation noise components $\left(v_{k}^{\xi^{i}},v_{t}^{\eta^{i}}\right)$ are assumed to have Gaussian distribution N(0,0.02), which roughly approximates the sonar precision equal to $1^{{}^{\circ}}$.

Note that, due to the port-starboard ambiguity, the bearing observations (4) do not allow an explicit inverse transformation to calculate the target coordinates, even in the absence of the noise $\left(v_{k}^{\xi^{i}},v_{t}^{\eta^{i}}\right)$. Nevertheless, the CMNF algorithm allows estimating $\left\{X_{k}\right\}$ with relatively high precision. First, the algorithm relates to the Bayesian estimator class, which allows for neutralizing the ambiguity not having an individual observer but the site as in the presented example. Second, the CMNF allows for fusing optimally the original observations and some of their transforms, such as least squares (LS) estimates or other regression technique results. An alternative way is not to rely entirely on the current measurements of the target position, but also to take into account some pre-collected data of such observations. This can be done by the aids of one of the machine learning (ML) regression algorithms, e.g., lasso regression [51]. This can provide not only the estimation quality gain, but also significantly reduce the amount of time necessary for the online measurements processing. While the LS method requires a nonlinear optimization problem solution for each observation round, an ML regression algorithm can provide much faster predictions with all the resource and time demanding procedures of the model fit being performed a priori. For instance, the lasso regression coefficients calculation also requires a nonlinear optimization problem solution and the whole training set processing, but, after that, the target position estimate is easily computed as a polynomial of the measurements.

**Figure 3:**
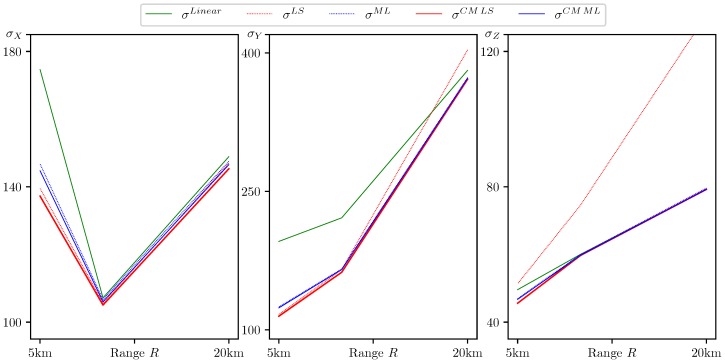
Coordinate-wise std of estimation error $\sigma^{E}$ for E∈{Linear,LS,ML,CMLS,CMML}.

In Table 1 and Figure 3, we present the standard deviations of the estimation errors for the following estimators:

- Linear: the basic minimax estimator (9), which uses only the original bearing measurements $Y^{b}=\mathrm{col}\left(\left\{\left(\xi^{i},\eta^{i}\right)\right\}\right)$, defined by relations (7). It is also the best linear estimate.
- LS: Linear squares estimate ${\hat{X}}^{LS}={\hat{X}}^{LS}\left(Y^{b}\right)$, calculated using the eight pairs of bearing measurements.
- ML: Lasso regression estimate ${\hat{X}}^{ML}={\hat{X}}^{ML}\left(Y^{b}\right)$, which uses normalized bearing measurements and their squares as features.
- CM LS: Minimax estimator (9) with original bearing measurements supplemented by the LS estimate: $Y^{LS}=\mathrm{col}\left(\left\{\left(\xi^{i},\eta^{i}\right)\right\}\cup {\hat{X}}^{LS}\left(Y^{b}\right)\right)$.
- CM ML: Minimax estimator (9) with original bearing measurements supplemented by the ML estimate: $Y^{ML}=\mathrm{col}\left(\left\{\left(\xi^{i},\eta^{i}\right)\right\}\cup {\hat{X}}^{ML}\left(Y^{b}\right)\right)$.

The covariances $R^{XY}$, which are necessary for the minimax estimates calculation with Formula (9), for the Linear, CM LS, and CM ML estimators, were calculated on the test set of $10^{6}$ position-measurement pairs by Monte Carlo sampling. A part of this test set was also used to fit the lasso regression model for estimators ML and CM ML. The standard deviations of the estimation errors were estimated on a different test set of the same size of $10^{6}$ samples.

From the results presented in Table 1 and Figure 3, one can conclude the following:

- The CM LS estimate uniformly outperforms all others (Table 1, in bold).
- All the presented algorithms of CM-family are able to neutralize the port-starboard ambiguity.
- The error standard deviations computed for the Linear and CM LS/ML estimators confirm the conclusion of Section 3.1. Indeed, the introduction of the additional measurements’ transform increased the performance of the minimax estimate in comparison with the best linear estimate. The last is true even for the case of $y_{0}=20\times 10^{3}$, where the LS estimate demonstrated very poor quality.
- The difference in the results for various distances between the target and the observers shows that the most benefit the minimax estimate can provide when the model at hand is far from linear. Indeed, for $y_{0}=20\times 10^{3}$, the difference between the estimate error standard deviations for linear and nonlinear minimax estimates is negligible, which is not the case for $y_{0}=5\times 10^{3}$, where the nonlinearity of the system is more pronounced.

### 4.2. Tracking of Maneuvering Target Given the Bearing-Doppler Multisensor Observations

In this section, we present the results of numerical simulation of a maneuvering target motion model (1)–(3) given the bearing-only (7) and bearing-Doppler observations (8).

The planar target motion in the target coordinate system 0xyz is defined by the SDS (1) with the acceleration equation parameters λ=0.01, ν=0.0 and μ=0.01. The initial conditions are:

$$\left(x_{0},y_{0},z_{0}\right)=\left(0,0,0\right),\;v_{0}∼R\left[5,12\right],\;\phi_{0}∼N\left(-\pi /2,0.1\right),\;a_{0}^{n}∼R\left[-0.2,0.2\right].$$

Here, the parameters to the Gaussian distribution N(m,σ) are the mean and standard deviation in meters, and R[a,b] is the uniform distribution in the interval [a,b]. The random rotation and shift parameters for the target coordinate system are as follows:

$$\alpha ,\;\beta ∼N\left(0,\pi /36\right),\;R_{0}^{X}∼N\left(0,10^{3}\right),\;R_{0}^{Y}∼N\left(20\times 10^{3},10^{3}\right),\;R_{0}^{Z}∼N\left(-10^{3},10^{2}\right).$$

It should be noted that the introduced initial conditions for the coordinates in the target coordinate system $x_{0}={\left(x_{0},y_{0},z_{0}\right)}^{T}$ and the shift $R^{0}={\left(R_{0}^{X},R_{0}^{Y},R_{0}^{Z}\right)}^{T}$ agree with the static model (13) for $y_{0}=20\times 10^{3}$.

**Figure 4:**
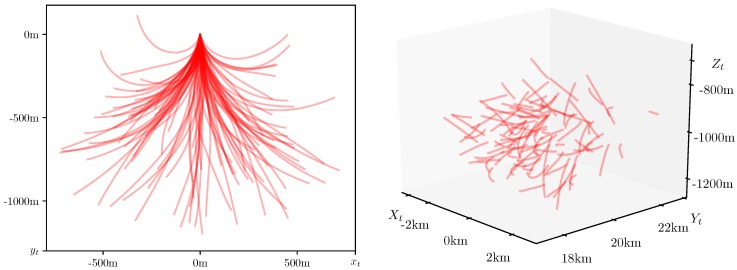
Sample paths in the target coordinate system 0xyz (**left figure**) and in the observer coordinate system 0XYZ (**right figure**).

In Figure 4, we demonstrate 100 sample paths of the system (1)–(3) with the parameters defined above. On the left, for clarity, we present the paths lying on the maneuver plane 0xy and starting from the common initial position. On the right, the same paths, being distributed according to the random initial position, are pictured in the observer coordinate system 0XYZ. Even though the model has only one scalar disturbing process $W_{t}$ in the equation for the normal acceleration $a_{t}^{n}$ in SDS (1), one can notice a rather wide variety of trajectories produced by the SDS with chosen parameters. At the same time, all the presented paths demonstrate smoothness, which is characteristic of the underwater environment.

The registration devices are also located at the same points (14) as it was assumed for the static case and have the same parameters of the noises in the bearing observations. For the Doppler measurements (5), the sound speed is set to C=1500 m/s, and the source frequency $\omega_{0}=20$ Hz. The noise in frequency measurements is also assumed to be Gaussian: $v_{k}^{\omega }∼N\left(0,0.005\right)$. Notably, the parameters of both the target motion and sonar accuracy are chosen similarly to those of the classic paper [23].

In this simulation study, we have compared the standard deviation of the estimate error for the following filters:

- LS: Linear squares estimate ${\hat{X}}_{k}^{LS}={\hat{X}}_{k}^{LS}\left(Y_{k}^{b}\right)$, calculated using the eight pairs of bearing measurements $Y_{k}^{b}=\mathrm{col}\left(\left\{\left(\xi_{k}^{i},\eta_{k}^{i}\right)\right\}\right)$. The same as in the static case, but applied along the whole path.
- ML: Lasso regression estimate ${\hat{X}}_{k}^{ML}={\hat{X}}_{k}^{ML}\left(Y_{k}^{b}\right)$, which uses normalized bearing measurements and their squares as features. The same as in the static case, but applied along the whole path.
- Basic CMNF: the conditionally minimax nonlinear filter (11) and (12), which uses the combined bearing and Doppler measurements $Y_{k}^{bd}=\mathrm{col}(\left\{\left(\xi_{k}^{i},\eta_{k}^{i},\omega_{k}^{i}\right)\right\}$, defined by (8).
- CMNF LS: the conditionally minimax nonlinear filter (11) and (12), which uses the original bearing measurements and the LS estimate $Y_{k}^{LS}=\mathrm{col}\left(\left\{\left(\xi_{k}^{i},\eta_{k}^{i}\right)\right\}\cup {\hat{X}}_{k}^{LS}\left(Y_{k}^{b}\right)\right)$.
- CMNF LS with Doppler: the conditionally minimax nonlinear filter (11) and (12), which uses the combined measurements and the LS estimate $Y_{k}^{LS}=\mathrm{col}\left(\left\{\left(\xi_{k}^{i},\eta_{k}^{i},\omega_{k}^{i}\right)\right\}\cup {\hat{X}}_{k}^{LS}\left(Y_{k}^{b}\right)\right)$.
- CMNF ML: the conditionally minimax nonlinear filter (11) and (12), which uses the original bearing measurements and the lasso estimate $Y_{k}^{ML}=\mathrm{col}\left(\left\{\left(\xi_{k}^{i},\eta_{k}^{i}\right)\right\}\cup {\hat{X}}_{k}^{ML}\left(Y_{k}^{b}\right)\right)$.
- CMNF ML with Doppler: the conditionally minimax nonlinear filter (11) and (12), which uses the combined measurements and the ML estimate $Y_{k}^{ML}=\mathrm{col}\left(\left\{\left(\xi_{k}^{i},\eta_{k}^{i},\omega_{k}^{i}\right)\right\}\cup {\hat{X}}_{k}^{ML}\left(Y_{k}^{b}\right)\right)$.
- A priori estimate: equal to the mean $X_{t}$. The standard deviation of this “trivial” estimate can be used as a measure of other filters’ performance: if a filter has std larger then that of a “trivial” estimate, then one can conclude that this filter diverges; if a filter’s std is much less than it deserves further analysis and application.

The model is simulated on the time interval t∈[0,100] s with discretization step Δ=1 s. The size of the training set for the CMNF filters, as well as of the test set, is equal to $10^{5}$ sample paths.

It should be noted that the presented model turns out to be quite challenging for EKF and UKF filters, which are common to apply when dealing with such nonlinear systems. Even in milder conditions, when only the planar motion is considered, and, along with the bearing observations, the distance is available; both mentioned filters demonstrate divergence [37].

Table 2 presents the standard deviations of the estimation errors for all the mentioned filters on the last step, which corresponds to the end of the observation interval T=100 s.

**Figure 5:**
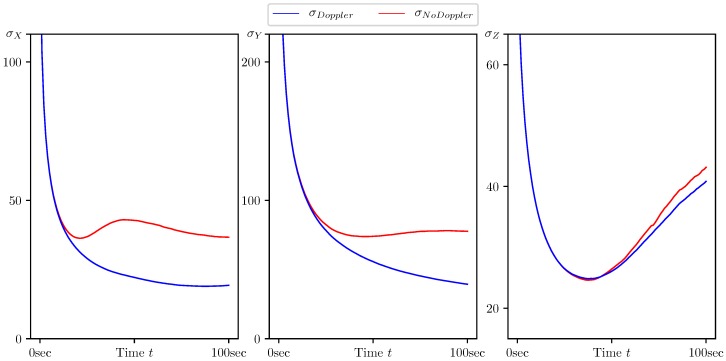
Standard deviation of estimation error for the coordinates $\left(X_{t},Y_{t},Z_{t}\right)$ calculated by CMNF filter with and without Doppler measurements.

**Figure 6:**
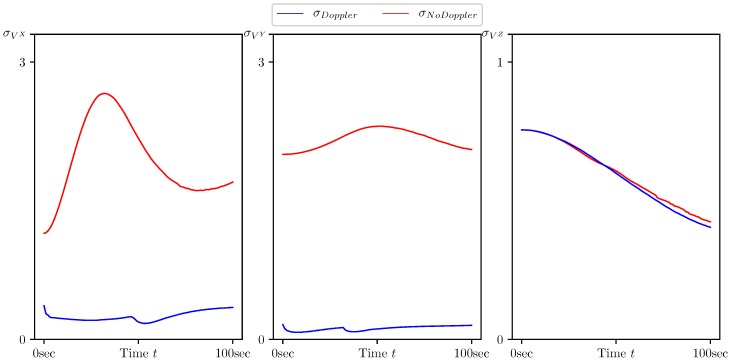
Standard deviation of estimation error for the target velocity components $\left(V_{t}^{X},V_{t}^{Y},V_{t}^{Z}\right)$ calculated by CMNF filter with and without Doppler measurements.

**Figure 7:**
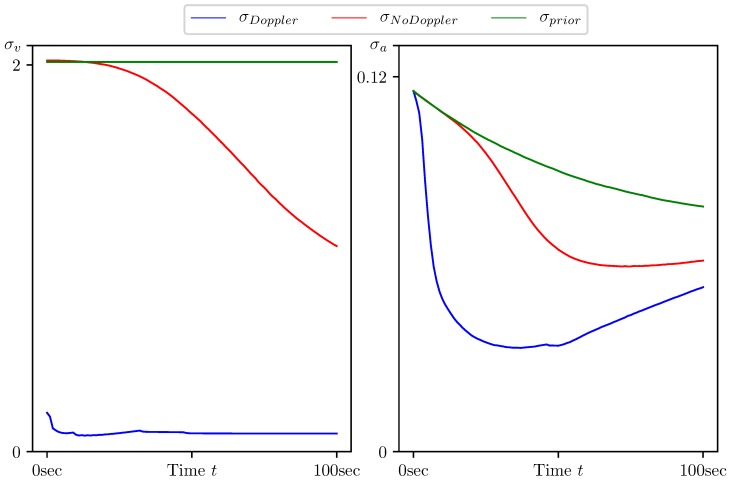
Standard deviation of estimation error for the target heading speed and normal acceleration $\left(v_{t},a_{t}^{n}\right)$ calculated by CMNF filter with and without Doppler measurements, and their a priori estimates.

**Figure 8:**
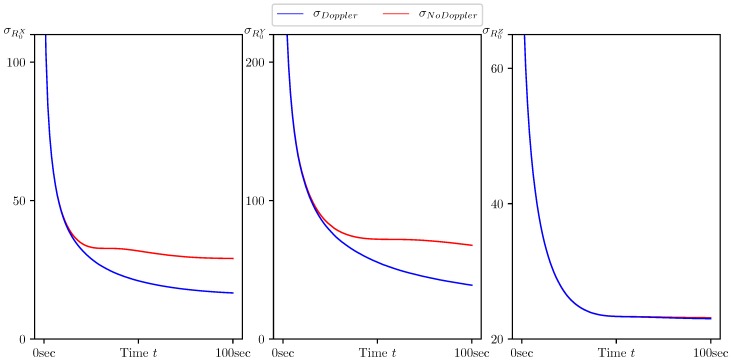
Standard deviation of estimation error for the target coordinate system shift $R^{0}={\left(R_{0}^{X},R_{0}^{Y},R_{0}^{Z}\right)}^{T}$ calculated by CMNF filter with and without Doppler measurements.

**Figure 9:**
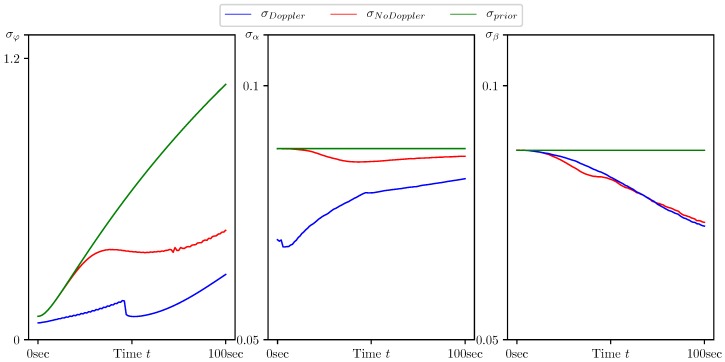
Standard deviation of estimation error for the target heading and coordinate system rotation angles ${\left(\varphi_{t},\alpha ,\beta \right)}^{T}$ calculated by the CMNF filter with and without Doppler measurements, and their a priori estimates.

In Figure 5, Figure 6, Figure 7, Figure 8 and Figure 9, the standard deviations for the parameter estimation errors are presented with respect to the time variable t∈[0,T], T=100 s. Table 1 shows little or no difference between the results for Basic CMNF, CMNF LS Doppler, CMNF ML Doppler filters, and between the results for CMNF LS and CMNF ML filters on the last step. The analysis of the collected data shows that all CMNF filters with Doppler measurements demonstrate almost the same behavior at all other time instants as well. Thus, in the following figures, the Basic CMNF, CMNF LS Doppler, and CMNF ML Doppler estimation error standard deviations are shown by the same line $\sigma_{Doppler}$. The same is true for the CMNF filters based on the bearing observations only, so the CMNF LS and CMNF ML estimation error standard deviations are also depicted by the same line $\sigma_{NoDoppler}$.

Figure 5 shows the superiority of the CMNF filters for the target coordinate estimation in comparison with all the static estimates presented in the Section 4.1. Together with Figure 6, it also provides evidence of a great performance boost provided by the Doppler measurements.

Figure 7 and Figure 8 demonstrate that, along with the target motion parameters, the parameters of its maneuver, namely the heading speed $v_{t}$ and normal acceleration $a_{t}$, can be estimated with a quality much better than one of the a priori estimate. The same is true for the target coordinate system shift $R^{0}$ since the a priori estimates standard deviations are equal to $\sigma_{prior}={\left(1000,1000,100\right)}^{T}$.

In contrast, Figure 9 shows rather poor performance for all the filters for the estimation of the angles α and β: the quality is almost the same as the quality of the estimate given by the mean value $\hat{\alpha }=\hat{\beta }=0$. At the same time, the standard deviation of the heading angle error $\varphi_{t}$ is notably less than that of the “trivial” estimate ${\hat{\varphi }}_{t}=E\left\{\varphi_{t}\right\}$.

The results presented in Table 2 and Figure 5, Figure 6, Figure 7, Figure 8 and Figure 9 show the following:

- The conditionally minimax nonlinear filter provides much better estimates for the target coordinates than the static ones, which do not take into account the information provided by the prediction “by virtue” of the system.
- All the presented algorithms of CM-family are able to neutralize the port-starboard ambiguity.
- Doppler measurements give a significant performance boost for CMNF estimates not only for the velocity parameters, but also for the target coordinates and normal acceleration.
- In contrast with the static case, where the introduction of the additional measurements’ transform significantly increases the performance of the estimate, here, the improvement can’t be called dramatic.

## 5. Conclusions

In the paper, we presented the results of the CMNF algorithm applied to the solution of the maneuvering AUV tracking problem, given the combination of the bearing-Doppler measurements. The measurement structure admitted port-starboard ambiguity. The mathematical model of the target movement represented a stochastic coordinate turn with random initial parameters: maneuver plane position, target heading speed, etc. The total dimensionality of the dynamic system state was 17.

The obtained estimates of the target state, i.e., the coordinates and speed, were unbiased and had high precision. The real estimation error covariance matrix was adequate to the corresponding theoretical values. The port-starboard ambiguity was neutralized. We showed the impact of the Doppler speed measurements on the AUV state estimation performance. It should be noted that the CMNF algorithm, being applied to the bearing-Doppler measurements, allowed us to estimate the initial parameters of the maneuver mentioned above.

We consider the following directions of further research in the same area. First, the optimization of the observations transform is a possible way to the estimate enhancement. Second, the optimization problem of the registration devices configuration and positioning looks promising. Third, the application of the obtained results for the identification of the target maneuver change and successive tracking of the AUV, performing a sequence of maneuvers, is practically important. The research on the mentioned subjects is in progress.

## Figures and Tables

**Table 1:** Coordinate-wise std of estimation error $\sigma^{E}$ for E∈{Linear,LS,ML,CM LS,CM ML}.

	$y_{0}=5\times 10^{3}$			$y_{0}=10\times 10^{3}$			$y_{0}=20\times 10^{3}$		
	$\sigma_{X}$	$\sigma_{Y}$	$\sigma_{Z}$	$\sigma_{X}$	$\sigma_{Y}$	$\sigma_{Z}$	$\sigma_{X}$	$\sigma_{Y}$	$\sigma_{Z}$
Linear	174.75	195.99	49.57	107.18	221.11	60.02	148.89	381.16	79.23
LS	139.57	116.69	51.31	105.62	165.49	74.61	146.97	403.34	130.72
ML	146.17	125.50	46.91	106.83	166.12	60.03	147.50	373.37	79.60
CM LS	137.28	114.79	45.56	105.04	162.44	59.69	145.30	371.56	79.22
CM ML	144.33	123.98	46.71	105.98	165.50	59.75	146.53	372.62	79.22

**Table 2:** Parameter-wise standard deviation of estimation error $\sigma^{F}\left(T\right)$ for F∈ {LS, ML, Basic CMNF (Doppler), CMNF LS (Doppler), CMNF ML (Doppler), a priori}.

	$\sigma_{X}\left(T\right)$	$\sigma_{Y}\left(T\right)$	$\sigma_{Z}\left(T\right)$	$\sigma_{V^{X}}\left(T\right)$	$\sigma_{V^{Y}}\left(T\right)$	$\sigma_{V^{Z}}\left(T\right)$	$\sigma_{v}\left(T\right)$	$\sigma_{a}\left(T\right)$	$\sigma_{\varphi }\left(T\right)$
LS	143.36	382.62	127.03	—	—	—	—	—	—
ML	148.62	362.73	89.55	—	—	—	—	—	—
Basic CMNF	19.30	39.37	40.84	0.345	0.152	0.404	0.094	0.053	0.278
CMNF LS	36.67	77.61	43.15	1.701	2.054	0.424	1.063	0.061	0.467
CMNF LS Doppler	19.30	39.37	40.83	0.345	0.152	0.404	0.094	0.053	0.278
CMNF ML	36.66	77.60	43.11	1.702	2.052	0.423	1.062	0.061	0.464
CMNF ML Doppler	19.30	39.36	40.82	0.345	0.152	0.404	0.094	0.053	0.278
a priori	—	—	—	—	—	—	2.0	0.115	0.1

## Abbreviations

The following abbreviations are used in this manuscript:

AUV	Autonomous underwater vehicle
CM	Conditionally minimax
CMNF	Conditionally minimax nonlinear filter
EKF	Extended Kalman filter
KF	Kalman filter
LS	Least squares
LKF	Linearized Kalman filter
ML	Machine learning
MS	Mean square
SDS	Stochastic differential system
UKF	Unscented Kalman filter
UT	Unscented transform
